# Macromolecular complexes in crystals and solutions

**DOI:** 10.1107/S0907444911007232

**Published:** 2011-03-18

**Authors:** Evgeny Krissinel

**Affiliations:** a CCP4, Research Complex at Harwell, Rutherford Appleton Laboratory, Harwell Science and Innovation Campus, Didcot, Oxon OX11 0FA, England

**Keywords:** macromolecular complexes, crystal packing, interactions

## Abstract

This paper presents a discussion of existing methods for the analysis of macromolecular interactions and complexes in crystal packing. Typical situations and conditions where wrong answers may be obtained in the course of ordinary procedures are presented and discussed. The more general question of what the relationship is between natural (in-solvent) and crystallized assemblies is discussed and researched. A computational analysis suggests that weak interactions with *K*_d_ ≥ 100 µ*M* have a considerable chance of being lost during the course of crystallization. In such instances, crystal packing misrepresents macromolecular complexes and interactions. For as many as 20% of protein dimers in the PDB the likelihood of misrepresentation is estimated to be higher than 50%. Given that weak macromolecular interactions play an important role in many biochemical processes, these results suggest that a complementary noncrystallographic study should be always conducted when inferring structural aspects of weakly bound complexes.

## Abstract 2

Methods for the analysis of the relationship between macromolecular complexes and interactions and their manifestation in crystal packing are described and discussed.

## 1. Introduction

Macromolecular crystallography is widely recognized as a major source of structural data on macromolecules and the interactions between them (Blundell & Johnson, 1976 ▶). It is often assumed that a biologically relevant interaction will manifest itself in crystal packing as a ‘significant’ interface that is indentifiable as such among other contacts between macromolecules in the crystal. In this context, ‘significance’ refers mostly to binding properties of the interface. Related to this is also a problem of the identification of macromolecular complexes in crystal packing. Assuming that a macromolecular complex does not change during crystallization, one can consider that all interfaces between monomeric units in the complex are binding and therefore ‘significant’. The complex structure may then be obtained by breaking (‘disengaging’) all other ‘insignificant’ crystal contacts.

**Figure 1:**
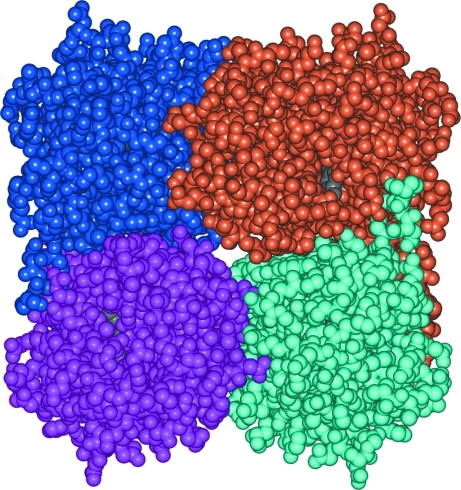
Homotetrameric complex of far-red fluorescent mKate protein easily identifiable by visual inspection of crystal packing (PDB entry 3bxc). However, the protein is found to be monomeric in solution (Pletnev *et al.*, 2008 ▶). The picture was produced using the *CCP*4*mg* graphical viewer (Potterton *et al.*, 2004 ▶).

In practice, the identification of significant interfaces and complexes is often performed by visual inspection of the crystal structure and matching the results with findings from com­plementary studies such as mass spectroscopy (Dass, 2001 ▶), NMR (Cavanagh *et al.*, 1996 ▶), electron microscopy (Frank, 2006 ▶) and scattering techniques (Feigin & Svergun, 1987 ▶; Svergun & Koch, 2002 ▶) as well as common biochemical evidence of binding properties (Berg *et al.*, 2002 ▶). This approach relies on the experience of the researcher and the availability of techniques for complementing experimental studies, as well as preliminary knowledge of the structure. As shown in some studies, visual inspection alone may lead to erroneous conclusions. For example, Fig. 1 ▶ shows a reasonably well packed homotetrameric complex which is easily identifiable in the crystal packing (PDB entry 3bxc). However, the structure has been found to be monomeric in solution (Pletnev *et al.*, 2008 ▶).

Automatic identification of macromolecular interactions and complexes from crystal packing has proved to be a challenging problem which does not have an ultimate solution to date. A number of approaches to the problem, ranging from bio­informatics to computational chemistry, have been reported in the literature. One of the commonly used rules of thumb is that a significant biologically relevant interface will manifest itself in different crystal forms and thus may be used for identification. This hypothesis was thoroughly investigated by Xu *et al.* (2008 ▶), who showed that it does work but is not without limitations. Useful suggestions may be obtained from com­parative homology analysis (Ogmen *et al.*, 2005 ▶). Con­siderable effort has been expended in attempts to assess the significance of an interface from its properties (see, for example, Argos, 1988 ▶; Miller, 1989 ▶; Janin & Chothia, 1990 ▶; Jones & Thornton, 1995 ▶, 1996 ▶; Tsai *et al.*, 1996 ▶; Lo Conte *et al.*, 1999 ▶; Ponstingl *et al.*, 2000 ▶; Chakrabarti & Janin, 2002 ▶; Gutteridge *et al.*, 2003 ▶). However, it was observed by Jones & Thornton (1996 ▶) that no uniform measure of interface significance may be derived for all complex types. A possible explanation of this fact was offered by Krissinel & Henrick (2007 ▶), who pointed out that interface properties should be evaluated with respect to the particular biochemical context. The significance of the interface is closely linked to its role in complex formation, a process which may be seen as a com­promise between binding (enthalpy change) and entropy loss. The enthalpic component, or internal energy of the complex, may be represented by interface properties. However, the entropy change depends on the complex size and geometry, and thus examining only interface properties is not sufficient for robust conclusions.

A few applications for automatic inference of macromolecular complexes and interactions from crystal packing have been developed to date. *PQS* [*Protein Quaternary Structure*, a web service at the European Bioinformatics Institute (EBI); Henrick & Thornton, 1998 ▶] attempts to identify significant interfaces using a buried surface-area measure. Having identified the significant interfaces, the macromolecular complex is built starting from a single chain by the progressive addition of suitable interfaces.

The *PITA* software (*Protein InTeractions and Assemblies*; Ponstingl *et al.*, 2003 ▶) exploits the idea of significant interfaces in a different way. In this method, the measure of significance is derived from statistical analysis of atom contacts in macromolecular interfaces as described by Ponstingl *et al.* (2000 ▶). In contrast to *PQS*, *PITA* constructs macromolecular complexes starting from the largest assembly allowed by the crystal structure. Making use of iterative bisectioning, the initial assembly is split into smaller complexes such that a combined score of engaged (belonging to a complex) interfaces achieves a certain threshold value.

*PISA* (*Protein Interfaces, Surfaces and Assemblies*; Krissinel & Henrick, 2007 ▶) is built on principles that differ from those used in *PQS* and *PITA*. In *PISA*, macromolecular complexes are identified as chemically stable associations, *i.e.* those with a positive free energy of dissocation. Using a graph-theoretical approach, *PISA* enumerates all assemblies that may potentially be formed in a given crystal packing and checks each one for chemical stability. Then, using a set of semiempirical rules, suitable candidates are ranged by their likelihood of being a correct answer.

Neither *PQS*, *PITA* nor *PISA* give absolutely correct identifications of complexes in crystal packing. The success rates of these methods are difficult to compare owing to the relatively low number of macromolecular complexes in the PDB that have solid independent (noncrystallographic) evidence for their three-dimensional structure. Thus, 218 nonredundant PDB entries with data that are ‘beyond doubt’ on their multimeric states were identified by Ponstingl *et al.* (2000 ▶) and were then used by Ponstingl *et al.* (2003 ▶) and Krissinel & Henrick (2007 ▶) for calibration and assessment purposes. Since 2007, *PISA* has been used as a major tool to aid quaternary-structure annotation in the PDB and although not all *PISA* predictions are automatically accepted, there is a possibility of developing a bias in favour of *PISA* results, especially in cases where depositors do not supply the PDB with data on the oligomeric state of their structures.

As a software application and web service, *PISA* is a reasonably convenient tool to use. Feedback from PDB and PDBe (PDB Europe, formerly known as the Macromolecular Structure Database at the EBI) curators, who use *PISA* on 20–­30 new entries daily, suggests that in 90–95% of instances *PISA* predictions coincide with experimental findings or, if those are not available, with what would be assigned to the entry using existing expertise, common sense and intuition. This picture gives an impression of *PISA* as a very reliable piece of software, which may result in overestimation of its limits and develop a temptation to shortcut on the additional experimentation needed for the verification of crystallo­graphic results. In this paper, we present an overview of typical situations where *PISA* does not give correct answers and suggest ways to interpret its results. We also discuss the relationship between natural and crystallized complexes and the conditions under which biologically relevant interactions may be misrepresented by crystal packing. In such cases, neither of the automatic tools is expected to deliver trustworthy results and complementary experimental evidence must be sought.

## 2. General background

The identification of macromolecular complexes in *PISA* is based on the evaluation of their free Gibbs energy of dissociation, 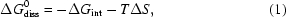
where Δ*G*_int_ represents the enthalpy of engaged macromolecular interfaces (binding energy), *T* is the temperature and Δ*S* is the entropic cost of dissociation. Where entropic cost prevails (Δ*G*_0_^diss^ < 0) the complex is driven towards the dissociated state. Therefore, complexes with positive Δ*G*_0_^diss^ are considered as chemically stable. Note that this definition of a stable complex does not imply that the equilibrium complex concentration is always higher than the concentration of its subunits (which can be either separate macromolecules or smaller complexes). Indeed, the equation of chemical equilibrium Δ*G*_0_^diss^ = *RT*log*K*_d_ = 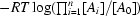 suggests that the complex concentration [*A*_0_] becomes higher than the subunit concentration [*A*_*i*_] at Δ*G*_0_^diss^ ≥ −(*n* − 1)*RT*log[*A*_*i*_]. For dimers (*n* = 2) at a typical [*A*_*i*_] of 1 m*M* this gives Δ*G*_0_^diss^ ≥ 4.1 kcal mol^−1^ (1 cal = 4.186 J).

A multimeric complex may dissociate in a number of ways or have more than one dissociation pattern, *e.g.* a homohexamer may dissociate into six monomers, three dimers or two trimers. The preferred dissociation pattern is identified as that with the lowest Δ*G*_0_^diss^ and may be found by analysing all possible dissociation scenarios. In (1), Δ*G*_int_ is calculated as the sum of the binding energies of the interfaces that are disengaged in a particular dissociation scenario. If long-range electrostatic interactions between dissociating sub­units may be neglected, Δ*G*_int_ may be estimated using only interface properties such as interface area, the chemical composition of the interface, hydrogen-bond and salt-bridge patterns *etc*. In contrast to Δ*G*_diss_, the entropic cost Δ*S* does not depend on the binding properties of interfaces. As shown in Krissinel & Henrick (2007 ▶), Δ*S* depends mostly on the number of dissociated subunits, their masses, shapes (through their moments of inertia) and symmetry properties. A minor contribution to Δ*S* from interface areas arises from the immobilization of flexible surface features in the associated state. An important contribution to Δ*S* comes from the change in the low-frequency vibration motion of subunits upon the formation of a complex (this contribution is difficult to estimate and is neglected in the *PISA* software). As a result, the free energy of dissociation Δ*G*_0_^diss^ cannot be represented as a function of individual interfaces unless severe approximations are applied.

Not every fragment of crystal packing may represent a potential complex. The graph-theoretical procedure developed by Krissinel & Henrick (2007 ▶) calculates a comprehensive list of formally correct ways to split a crystal structure into complexes by disengaging different subsets of crystal interfaces. Here, formal correctness refers to symmetry considerations. *PISA* applies this procedure automatically and checks the obtained complexes for chemical stability according to (1). Only stable structures are left in the final list which is presented to the user.

**Figure 2:**
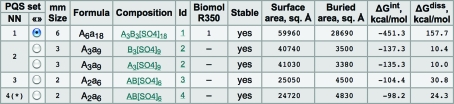
Protein complexes suggested by *PISA* for PDB entry 2h07 (snapshot from the *PISA* website; http://www.ebi.ac.uk/msd-srv/pisa/cgi-bin/piserver?qa=2h07). Each PQS set represents a way to split the crystal into complexes; *e.g.* the second solution corresponds to two trimers. These trimers are structurally similar (but not crystallographically identical), which is indicated by the assignment of the same ID to them. Δ*G*_0_^diss^ is given in the rightmost column. A detailed description of the data in the columns is obtainable from *PISA*’s online help by clicking on the column titles. See discussion in the text.

A typical example of *PISA* output is presented in Fig. 2 ▶. In this example, *PISA* suggests that there are four different ways to split the crystal into chemically stable complexes, which are represented by four PQS (probable quaternary structure) sets. In the first set the crystal is split into hexamers and in the second set into two trimers; the third and fourth solutions correspond to structurally different dimers. In the second set, the trimers are structurally similar (and therefore are assigned the same ID) but are not crystallographically identical. It is not always the case that a crystal is split into structurally similar complexes. A good example of the opposite is given by PDB entry 1e94 (Song *et al.*, 2000 ▶), which contains cocrystallized hexamers and dodecamers.

In order to come to a conclusion on a protein’s oligomeric state, a user would need to choose between the PQS sets but not between individual complexes within the sets (the solution to the problem is represented by the whole set rather than individual complexes). What choice should be rated as a correct choice? Obviously, one would want to identify the oligomeric state found in the protein’s native environment. However, sometimes this is not possible. In a number of instances the oligomeric state of a protein may be weakly defined, *i.e.* it may vary depending on the external conditions. Protein–protein complexes, which may dissociate or associate depending on the biochemical environment, play an important role in many biological processes, such as signal transduction (Gomperts *et al.*, 2002 ▶), electron transport (Brown *et al.*, 1999 ▶; Doyle *et al.*, 1986 ▶; Ren *et al.*, 1993 ▶), transcriptional regulation (Huang *et al.*, 1997 ▶; Sengchanthalangsy *et al.*, 1999 ▶), growth factors (Lu *et al.*, 1995 ▶; Hsu *et al.*, 1997 ▶; Bianchet *et al.*, 2000 ▶; Blundell *et al.*, 2000 ▶), molecular switches (Darling *et al.*, 2000 ▶; Pan & Heitman, 2002 ▶; Ma & Karplus, 1997 ▶), cell–cell recognition (Alattia *et al.*, 1997 ▶) and many others (Waas & Dalby, 2002 ▶; Cho *et al.*, 2006 ▶; Johannes, 2007 ▶; Bonet *et al.*, 2006 ▶; Ansari & Helms, 2005 ▶; Nooren & Thornton, 2003 ▶; Schnarr & Khosla, 2006 ▶; Vaynberg & Qin, 2006 ▶; Fuentes *et al.*, 2006 ▶, 2007 ▶; Boelens *et al.*, 1991 ▶; Ceres & Zlotnick, 2002 ▶; Buts *et al.*, 2001 ▶; Nyfeler *et al.*, 2005 ▶; Hamelryck *et al.*, 2000 ▶). The dissociation constant *K*_d_ of weak complexes may reach a few hundred µ*M* (Nooren & Thornton, 2003 ▶), which corresponds to a Δ*G*_diss_ of only a few kcal mol^−1^. Experimental identification of the structural features of such complexes is difficult because of their transient nature (see, for example, Vaynberg & Qin, 2006 ▶; Fuentes *et al.*, 2006 ▶, 2007 ▶; Buts *et al.*, 2001 ▶). One might think that in the course of the crystallization procedure weakly bound complexes have a chance of being sacrificed (disassembled) in favour of nonspecific contacts if this results in the formation of crystal packing that is more suitable from a global energy point of view. If this happens then the most significant crystal interface does not correspond to the biologically related interaction or, in other words, the interaction is misrepresented by the crystal packing. Although it was found in a number of studies that weak interactions may manifest themselves in highly condensed pre-crystal solutions and crystalline states (examples are given in Ren *et al.*, 1993 ▶; Bianchet *et al.*, 2000 ▶; Blundell *et al.*, 2000 ▶; Nooren & Thornton, 2003 ▶; Boelens *et al.*, 1991 ▶; Ceres & Zlotnick, 2002 ▶; Buts *et al.*, 2001 ▶; Hamelryck *et al.*, 2000 ▶), the overall probability of observing a weak biologically related interaction as a crystal interface remains unclear.

**Figure 3:**
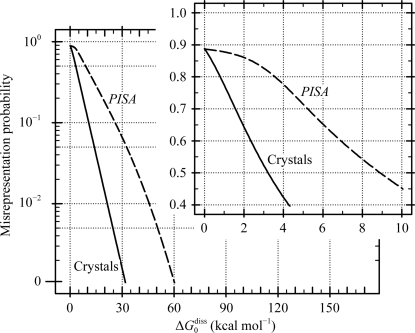
The probability of protein dimers being misrepresented by crystals (solid line) and in *PISA* analysis (dashed line) as a function of the dissociation free energy Δ*G*_0_^diss^. The estimates are obtained from the results of a computational docking study performed by Krissinel (2010 ▶). See text for details.

An attempt to shed light on the situation was performed by Krissinel (2010 ▶). In this study, a large ensemble of protein dimers were generated by a computational docking procedure and compared with the corresponding complexes inferred from crystal packing. The docking procedure looks for the optimal mutual arrangement of complex subunits in the absence of a crystal environment and therefore may be viewed as an approximation to the in-solvent situation. It was found that the probability of reproducing a crystal interface in docking experiments increases exponentially with increasing dissociation free energy Δ*G*_0_^diss^. From these results it proved to be possible to estimate the misrepresentation probability for dimers in *PISA* analysis. This probability is shown by a dashed line in Fig. 3 ▶. One can see two reasons why macromolecular complexes inferred from crystal packing are misrepresented (or, in simple words, differ from structures in a solvent environment). Firstly, this is a result of energy model approximations and computational errors in *PISA*; secondly, the complexes may be misrepresented by crystal packing as discussed above. Further mathematical analysis of docking results allowed the estimatation of pure crystal effects, the probability of which is shown by a solid line in Fig. 3 ▶ (Krissinel, 2010 ▶).

As seen from Fig. 3 ▶, weak interactions with Δ*G*_0_^diss^ ≤ 3–5 kcal mol^−1^ have a high probability of being lost during the course of crystallization. The crystal misrepresentation effect disappears completely at Δ*G*_0_^diss^ ≥ 30 kcal mol^−1^, *i.e.* when the complexes are bound by forces comparable with covalent linking or, in simple words, when the complexes are as stable as their monomeric units. As expected, errors in *PISA* are noticeably higher than estimated crystal effects. When in-crystal and in-solvent complexes are expected to always coincide (Δ*G*_0_^diss^ ≥ 30 kcal mol^−1^), *PISA* is likely to give 5–10% errors, which agrees with previous estimates (Krissinel & Henrick, 2007 ▶).

Fig. 3 ▶ suggests that the free energy of complex dissociation Δ*G*_0_^diss^ is a key parameter for the interpretation of *PISA* results. This value is reported in the rightmost column of Fig. 2 ▶. From the data presented in Fig. 2 ▶, the choice of the first solution, the hexamer, seems to be an obvious choice owing to the outstanding value of Δ*G*_0_^diss^ compared with the other alternatives. Indeed, according to the data in Fig. 3 ▶ Δ*G*_0_^diss^ ≃ 158 kcal mol^−1^ (the hexamer) corresponds to zero misrepresentation probability, while at Δ*G*_0_^diss^ ≃ 10 kcal mol^−1^ (the trimers) a 50% error is expected. This interpretation of *PISA* results would be quite naive. It is important to realise here that Δ*G*_0_^diss^ is not a ‘score’ to be interpreted as ‘the higher, the better’. For example, *PISA* suggests a hexamer with Δ*G*_0_^diss^ ≃ 19 kcal mol^−1^ as the first choice for PDB entry 2wyl (Garces *et al.*, 2010 ▶) and dimers with Δ*G*_0_^diss^ ≃ 47 kcal mol^−1^ as an alternative solution. According to the graph in Fig. 3 ▶ the dimers appear to be a considerably more reliable solution (∼20% chance of being an error for the hexamer *versus* ∼0.5% error probability for the dimers). However, it is the hexamer that should be chosen as the most probable multimeric state in this case. Indeed, analysis of the dissociation patterns (also given by *PISA*) reveals that the hexamer is a complex of three dimers which is thermodynamically stable (Δ*G*_0_^diss^ > 0), although less stable than the dimers. One can derive here a picture of dynamic equilibrium between the hexamer and dimers which is shifted toward the hexamer (*c.f.* the example in the beginning of this section). *PISA* assesses potential solutions (PQS sets) automatically in order to place the most probable one at the top of the list, using the following set of rules (ordered by priority).

(i) Higher multimeric states are preferred over lower states.
(ii) Solutions with a single type of complex are preferred over mixed-type solutions.
(iii) Complexes with a higher free energy of dissociation Δ*G*_0_^diss^ are preferred over those with a lower Δ*G*_0_^diss^.

As follows from the data in Fig. 3 ▶, interpretation of *PISA* results is difficult at low Δ*G*_0_^diss^, where they are very sensitive to the accuracy of energy calculations and may be influenced by crystal effects. There are not sufficient experimental data to give a reliable assessment of the energy-calculation errors in *PISA*, but limited evidence suggests a range of ±5 kcal mol^−1^. This error is comparable with that resulting from disregarding the specific chemical conditions (pH, concentration, ionic strength, salinity and temperature) in *PISA*. Therefore, in the case of low Δ*G*_0_^diss^ the final decision should always be confirmed by independent experimental data. For example, in the case of the pseudo-tetrameric 3bxc (*c.f.* Fig. 1 ▶) *PISA* reports Δ*G*_0_^diss^ ≃ 0.6 kcal mol^−1^, which is obviously on the edge of stability and this answer can be discarded. However, *PISA* also suggests relatively stable dimers with Δ*G*_0_^diss^ ≃ 12 kcal mol^−1^ in this case. The fact that these dimers are not reported in experimental studies may be accounted for by the combination of errors in *PISA* energy calculations and the specific chemical environment in the experimental setup.

Compared with the various scores used for the identification of multimeric states (Ponstingl *et al.*, 2003 ▶; Henrick & Thornton, 1998 ▶), the dissociation free energy has the advantage that it allows a wider interpretation of experimental results in chemical terms simply because chemical systems are driven by the free-energy function. If Δ*G*_0_^diss^ could be calculated with a well controlled accuracy, inferring macromolecular inter­actions and multimeric states from crystal packings would be a relatively straightforward procedure. However even then the devil is always in the details and in the next section we shall consider a few typical situations in which *PISA* predictions would not be correct even if Δ*G*_0_^diss^ were calculated precisely.

## 3. Where chemistry ‘makes no sense’

**Figure 4:**
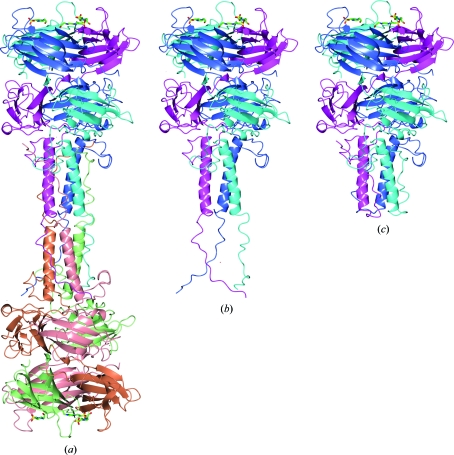
(*a*) Homohexamer predicted by *PISA* for PDB entry 1qex. (*b*) 1qex homotrimer identified as the biological unit in an experimental study by Rossmann *et al.* (2004 ▶); the hexamer is predicted to dissociate into two trimers. (*c*) Alternative homotrimer 1s2e obtained by different main-chain tracing in the same electron-density maps as 1qex; 1s2e trimers are correctly identified by *PISA* as not forming stable hexamers. See discussion in the text. The images were produced using the *CCP*4*mg* graphical viewer (Potterton *et al.*, 2004 ▶).

One of the most striking examples where the Δ*G*_0_^diss^-based procedure for automatic identification of macromolecular complexes grossly fails is given by PDB entry 1qex (bacterio­phage T4 gene product 9; Kostyuchenko *et al.*, 1999 ▶). This assembly is predicted to be a very stable homohexamer, shown in Fig. 4 ▶(*a*), with Δ*G*_0_^diss^ ≃ 104 kcal mol^−1^. According to the data in Fig. 3 ▶, there is zero probability that this structure is misrepresented by crystal packing. However, strong experimental evidence suggests that the complex is homotrimeric as shown in Fig. 4 ▶(*b*) (Rossmann *et al.*, 2004 ▶). The homotrimer represents the dissociation subunit of the 1qex hexamer and is also estimated by *PISA* to be very stable, with Δ*G*_0_^diss^ ≃ 90 kcal mol^−1^. Therefore, it appears that the predicted homohexamer is an error on a scale that is far beyond any reasonable range which needs to be explained.

According to experimental findings (Rossmann *et al.*, 2004 ▶), one biological function of 1qex homotrimers is to provide attachment of long-tail fibres to the T4 virus baseplate. The long-tail fibres represent elongated structures which mount and hold the virus on the cell membrane. The trimers are placed at the begining of the fibres and attach to the virus baseplate at variable angles with three short tails formed by N-­terminal domains of the polypeptide chains (seen in Fig. 4 ▶ *b* ▶). One could imagine that the attachment should be very strong for the trimers to serve as mounting elements for the long-tail fibres. From these considerations, it is not surprising that the engagement of two trimers with their N-terminal tails results in a highly stable hexameric complex and this engagement may take place during crystallization. However, this scenario does not explain why the N-terminal tails do not interact with each other in the trimer (the tails appear to be separated from each other in Fig. 4 ▶ *b*) and how the association of trimers is avoided during the course of virus assembly.

The most plausible answer to these questions was offered by one of the authors of the 1qex structure (Dr Sergey Strelkov, University of Leuven, Belgium; private communication at the ECM-23 meeting in 2006). It appears that electron-density maps of 1qex allow alternative tracing of the protein backbones in which the short tails of each 1qex trimer are replaced by the corresponding parts from its hexamer-forming partner. The alternative structure is represented by PDB entry 1s2e (Kostyuchenko *et al.*, 1999 ▶; shown in Fig. 4 ▶ *c*). In *PISA* analysis, 1s2e forms stable homotrimers that do not merge into hexamers. Therefore, it should be concluded that the homohexameric complex in Fig. 4 ▶(*a*) is an artifact resulting from an inappropriate interpretation of the electron-density maps.

**Figure 5:**
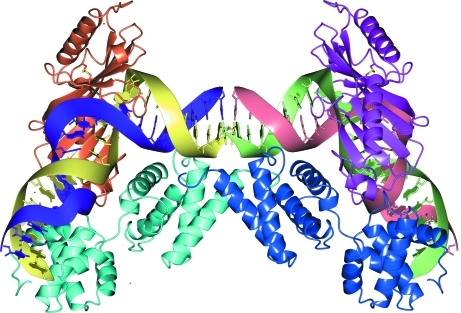
Heterooctamer predicted by *PISA* for PDB entry 1d3u. The complex dissociates into two identical heterotetramers forming the right-hand and left-hand parts of the octamer. It is the tetramers, rather than the octamer, that are identified as the biological units in this case (Littlefield *et al.*, 1999 ▶). See discussion in the text. The image was produced using the *CCP*4*mg* graphical viewer (Potterton *et al.*, 2004 ▶).

Another typical situation where *PISA* results need interpretation beyond straightforward chemical considerations is exemplified by PDB entry 1d3u. For this entry, *PISA* analysis suggests a heterooctameric complex (shown in Fig. 5 ▶). The octamer is reasonably stable, with Δ*G*_0_^diss^ ≃ 18 kcal mol^−1^, and is predicted to dissociate into two heterotetramers that form the left-hand and right-hand halves of the structure as shown in the figure. The tetramers appear to be somewhat less stable than the octamer (Δ*G*_0_^diss^ ≃ 14 kcal mol^−1^), yet they are the biological units here (Littlefield *et al.*, 1999 ▶).

A close examination of the complex and in particular the interface between the tetramers helps to reveal why a correct identification of multimeric complexes purely from first principles is not possible for 1d3u. This is because neither the octamer nor the tetramer represent natural assemblies. Indeed, for crystallization purposes the virtually infinite DNA strands are replaced by chemically synthesized 24-base fragments bound to protein parts of the complex. The removal of a large DNA section between the tetramers allows them to engage in a contact that, contrary to basic *PISA* assumptions, cannot occur under natural conditions. The resulting inter­action is purely artifactual, yet it appears to be substantial. As estimated by *PISA*, the interface between two contacting helices of the left-hand and right-hand tetramers in Fig. 5 ▶ shows a relatively high hydrophobic interaction of ∼−7.4 kcal mol^−1^ and forms 18 hydrogen bonds and eight salt bridges, which add a further ∼−14 kcal mol^−1^ to the interface binding energy Δ*G*_int_. In addition to this, the DNA fragments of the tetramers make a cross-pairing which involves three bases from each side. Examination of the DNA interface in Fig. 5 ▶ reveals a stacking interaction which adds about −9.5 kcal mol^−1^ to Δ*G*_int_. Combined together, these numbers indicate a mutual affinity of the tetramers, which in *PISA* estimates would be strong enough to merge them into octamers if the inter-tetramer contacts were not a mere artifact of the crystal packing.

**Figure 6:**
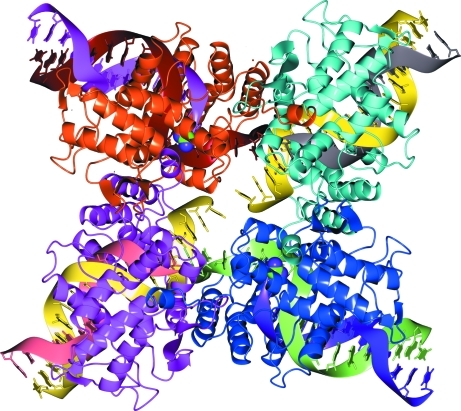
Heterododecamer predicted for PDB entry 1crx (DNA-recombination synapse). The complex is made of four heterotrimers, each including a DNA fragment bound to bacteriophage recombinase Cre. It is the trimers, rather than the dodecamer, that are considered as the primary units here (Guo *et al.*, 1997 ▶). See discussion in the text. The image was produced using the *CCP*4*mg* graphical viewer (Potterton *et al.*, 2004 ▶).

From a first glance, the same considerations should also apply to PDB entry 1crx (Guo *et al.*, 1997 ▶). For this entry, *PISA* analysis suggests a heterododecameric complex (shown in Fig. 6 ▶). The complex is made of four very similar but not identical heterotrimers. Each trimer includes a DNA fragment bound to bacteriophage recombinase Cre. The complex is predicted to dissociate at Δ*G*_0_^diss^ ≃ 28 kcal mol^−1^, giving two heterohexamers that make up the upper and lower halves of the structure as presented in Fig. 6 ▶. Just as in the case of 1d3u, the mutual arrangement of short DNA fragments seems to suggest rather clearly the artifactual nature of the intertrimer interfaces.

However, despite the superficial similarities between the complexes presented in Figs. 5 ▶ and 6 ▶ the latter represents a real complex: a site-specific DNA-recombination synapse (Guo *et al.*, 1997 ▶). The complex facilitates a three-stage DNA-recombination reaction which starts with opening the two DNA strands that run across the upper and lower hexamers in Fig. 6 ▶. In the next stages, the open strands are recombined one by one into strands running vertically through the left and right halves of the dodecameric structure in the figure. In fact, the crystallized structure may be viewed as a snapshot of the recombination machine with all DNA strands open. This particular state is indeed artificial in the sense that it was obtained using a representation of DNA in the form of short fragments. Nevertheless, in this case such a representation fits the context of the complex’s function and therefore the crystallized model of the complex appears to be valid. In the PDB the structure is annotated as heterohexameric, where the hexamer refers to both the upper and lower parts of Fig. 6 ▶ (these parts are identified by *PISA* as dissociation subunits). Apparently, this annotation refers to the Cre–LoxA complex, which pre-exists the formation of the recombination synapse and may be considered as a ‘more basic’ element for this (Guo *et al.*, 1997 ▶). This case demonstrates a situation in which the definition of the biological unit is to a certain degree subjective and cannot be completely algorithmic.

**Figure 7:**
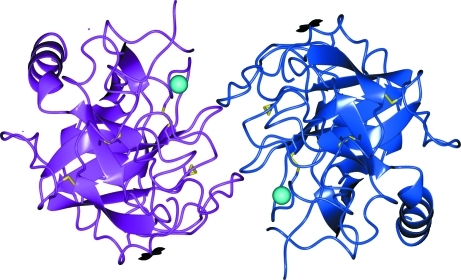
A stable dimeric complex predicted by *PISA* for PDB entry 1ton (tonin). Cyan spheres represent Zn^2+^ ions, which mediate the interation between monomeric chains. See discussion in the text. The image was produced using the *CCP*4*mg* graphical viewer (Potterton *et al.*, 2004 ▶).

Quite often, the interpretation of *PISA* results results in confusion because of the presence of ligands or small molecules and ions in crystals. For example, Fig. 7 ▶ presents a homodimeric complex predicted by *PISA* for PDB entry 1ton. The complex appears to be a rather stable one, with a dissociation free energy of Δ*G*_0_^diss^ ≃ 27 kcal mol^−1^. Visual inspection of the complex reveals that dimerization is substantially assisted by two Zn^2+^ ions (shown as cyan spheres in Fig. 7 ▶) which mediate the interface between the two tonin molecules. However, the ions are not part of the natural tonin structure. Instead, they were added to the crystallization buffer in order to stimulate the crystallization of tonin (Fujinaga & James, 1987 ▶). Therefore, the Zn ions should be excluded from the analysis. Re-examination of 1ton in *PISA* with the Zn ions removed from the entry shows a considerable decrease in the dissociation free energy to 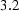
 kcal mol^−1^. This decrease is found to be in good agreement with the experimental estimate of about 16 kcal mol^−1^ for Zn–protein binding (DiTusa *et al.*, 2001 ▶). The resulting value of Δ*G*_0_^diss^ ≃ 3.2 kcal mol^−1^ is unspecific owing to the finite accuracy of energy calculations in *PISA* and the high probability of misrepresentation, as discussed in the previous section. Therefore, the predicted strong tonin dimer should be regarded as a clear artifact arising from the crystallization conditions and the protein is most probably monomeric. Indeed, this was confirmed experimentally (Fujinaga & James, 1987 ▶).

**Figure 8:**
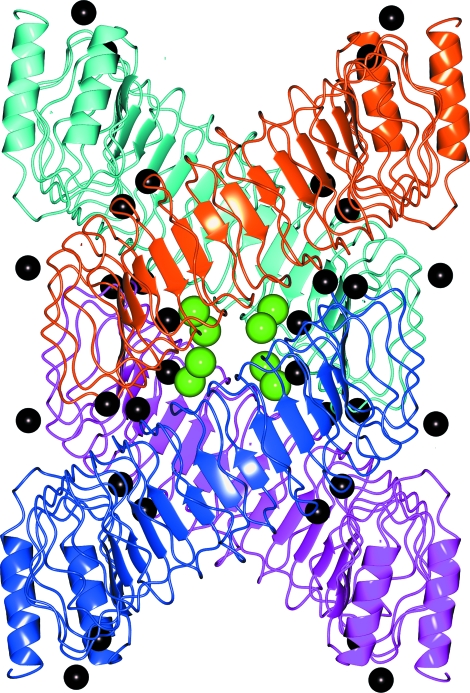
A stable homotetrameric complex predicted by *PISA* for PDB entry 1g9u (*Y. pestis* cytotoxin YopM). Green and black spheres represent Ca^2+^ and Hg^2+^ ions, respectively. The tetramer represents a superhelix featuring a hollow cylinder with an inner diameter of ∼35 Å. The same structure was obtained in a different crystal form: PDB entry 1jl5. See discussion in the text. The image was produced using the *CCP*4*mg* graphical viewer (Potterton *et al.*, 2004 ▶).

The presence of binding agents in crystals may be a serious obstacle to the interpretation of experimental results. A particular difficulty is met when, for example, zinc ions are part of the natural complex yet an additional Zn^2+^ concentration is added to the crystallization buffer. Sometimes, stimulated crystallization results in beautifully designed assemblies that look purposeful and imply a functional context. A clear example here is given by the homotetrameric assemblies inferred from PDB entries 1jl5 and 1g9u (Evdokimov *et al.*, 2001 ▶; shown in Fig. 8 ▶). Both entries represent the same protein, *Yersinia pestis* cytotoxin YopM, obtained in different crystal forms (*I*4_1_22 and *P*4_2_22 for 1jl5 and 1g9u, respectively). In both cases the protein packs into a superhelical structure described as a hollow cylinder with an inner diameter of ∼35 Å.

Generally speaking, the manifestation of interactions and structural features in different crystal forms is considered to be important evidence of their biochemical relevance (Xu *et al.*, 2008 ▶). However, *PISA* analysis suggests the that the 1jl5 and 1g9u tetramers are not chemically identical: the 1g9u tetramer appears to be a stable structure with Δ*G*_0_^diss^ ≃ 37 kcal mol^−1^, while its twin in 1jl5 appears to have a very weak association with a Δ*G*_0_^diss^ of only ∼3 kcal mol^−1^. Further analysis of *PISA* results revealed that this difference is a consequence of the twofold higher concentration of metal ions in 1g9u. From these figures, given that in both cases the addition of metal ions was essential for crystallization, one may suspect an artificial nature of the YopM tetramer. Indeed, additional experiments using size-exclusion chromatography and glutaraldehyde cross-linking suggested that the protein only undergoes oligomerization upon addition of calcium to the solution (Evdokimov *et al.*, 2001 ▶). In contrast, the removal of metal ions from the PDB entries results in the protein becoming monomeric in the *PISA* analysis as well, which is found to be in agreement with the experimental findings.

Ligand effects on energy calculations in *PISA* may be quite significant. For example, the removal of sulfate ions from PDB entry 2h07 (Li *et al.*, 2007 ▶) decreases the dissociation free energy of the hexamer in Fig. 2 ▶ from ∼158 to ∼36 kcal mol^−1^ and makes 2h07 trimers unstable. A conclusion that can be drawn from user feedback is that the neglect of ‘parasite’ protein–ligand interactions in crystal packing is by far the most common source of misinterpretation of *PISA* results. This situation can hardly be helped. It is not possible using only the data in a PDB file to decide reliably in automatic mode which ligands are native to the system in question and which ones represent pollutants and artificial additions such as precipitants added in order to aid crystallization. The interactive web server *PISA* allows a user to specify manually which ligands should be excluded from the analysis of oligomeric states. This option should be used each and every time non-native ligands are found in the entry.

## 4. Conclusions

The identification of macromolecular complexes in crystal packing is not a straightforward procedure, although it appears to have no particular complications in the majority of cases. It is important, however, to keep in mind that both computational methods and crystals provide us with models of biological macromolecules and their interactions and com­plexes rather than their precise representations. These models have a wide range of quality and trustworthiness. In general, a model is reliable if the effect of the crystal environment, in energy terms, is much smaller than the effect of possible variations within the model. From these considerations, covalently linked polypeptide chains with strong hydrophobic cores should be relatively stable structures that are insensitive to the difference between natural and experimental conditions, so that good models of them are expected. This fact is used implicitly by many robust methods in protein crystallo­graphy such as, for example, molecular replacement (Evans & McCoy, 2008 ▶). If a protein chain has low energy barriers to domain movement it may be crystallized in one of many possible conformations. A good example here is given by PDB entry 1oao, in which identical sequences are found in significantly different con­formations within the same asymmetric unit (Darnault *et al.*, 2003 ▶).

Macromolecular complexes may be considered as being similar to multi-domain protein chains, with the only difference being that unlike domains monomeric units in complexes are not covalently linked. If binding forces between the sub­units of a complex are comparable with covalent linking, the natural complex structure is likely to be preserved in the crystal, quite similarly to the case of domain packing discussed above. In such cases the identification of a complex is normally not a problem and very often can be performed by visual inspection. Weaker complexes, which are bound by forces comparable to those making the crystal lattice, are more difficult to identify. As demonstrated above, both software tools and visual examination may provide incorrect answers. In addition, weak complexes may be misrepresented by crystal packing, the probability of which is likely to grow exponentially with the decrease in the dissociation free energy of a complex. According to Krissinel (2010 ▶), as many as 19% of non­redundant dimers in today’s PDB may be misrepresented by crystal packing.

However, weakly bound associations and transient com­plexes play an important role in many biochemical processes and therefore are of considerable practical interest (*c.f.* the discussion and references in Krissinel, 2010 ▶). Admittedly, pure crystallographic evidence is often insufficient for reliable conclusions in such cases, and experimental results, whether processed by *PISA* or not, need to be complemented by independent noncrystallographic experimental studies. The estimate of misrepresentation probability shown in Fig. 3 ▶ suggests that automatic determination of even relatively tight complexes with PISA is not error-proof; therefore, it would be good practice to always confirm the oligomeric state of the protein in solution by experimental means.

In this study, we have discussed various situations in which automatic identification of macromolecular complexes from crystallographic data is difficult and results in confusion. In most cases the difficulty arises from the presence of crystallization agents, possible misrepresentation effects, modification of natural structures and ambiguity in the interpretation of electron-density maps. It was demonstrated that the dissociation free energy Δ*G*_0_^diss^ is a powerful, although not ultimate, indicator of the trustworthiness of *PISA* results. Where Δ*G*_0_^diss^ is low, as (approximately) quantified by the graph in Fig. 3 ▶, a validation study should be conducted similar to the research cases presented in this paper.
